# The complete mitochondrial genome of Korean indigenous stag beetle, *Dorcus koreanus*

**DOI:** 10.1080/23802359.2020.1835581

**Published:** 2020-11-11

**Authors:** Eunjoong Kim, Philjae Kim, Seung Lak An

**Affiliations:** a Research & Development Division, National Science Museum, Daejeon, Korea; b Division of Ecological Conservation, Bureau of Ecological Research, National Institute of Ecology, Choongnam, Korea

**Keywords:** Stag beetle, complete mitogenome, *Dorcus koreanus*, phylogenetic analysis

## Abstract

In this study, the mitogenome sequences of Korean indigenous stag beetle, *Dorcus koreanus*, was completely determined by next-generation sequencing analysis. The complete mitogenome of *D. koreanus* has 15,421 bp in length and consists of 13 protein-coding genes (PCGs), 22 tRNAs, two rRNAs, and one control region. The gene orders and content were identical with previously recorded mitogenomes of Lucanidae species. Phylogenetic analysis based on mitogenome dataset, consisting PCGs was revealed the taxonomical position in the family Lucanidae.

*Dorcus koreanus* Jang and Kawai, 2008 was newly described from Haenam of South Korea, based on morphological features in recently. The molecular taxonomical study of *D*. *koreanus* within related species was conducted by Han et al. (2010). However, this molecular research was performed using only one of partial gene sequence, and the complete genome informations of stag beetle including genus *Dorcus* were noticeably insufficient. Therefore, we provide the complete *D*. *koreanus* mitogenome sequences (MT984569) for both contributing the accurate establishment of evolutionary relationship of *Dorcus* and improving molecular information diversity of stag beetle.

The specimen was collected in Haenam, South Korea (34°28′35.04″N, 126°36′47.4″E) on 26 February 2020. The voucher specimens (NSMK-IN00001) and mitochondrial DNA (mt-DNA) sample (NSMK-DN00004) were stored in −80 °C of National Science Museum (Daejeon, Korea). Experiment steps for mitochondrial DNA analysis conformed to the method described by Kim et al (2020).

The complete mitogenome of *D. koreanus* (MT984569) is composed 15,421 bp of nucleotide. The genome contained of 13 PCGs, 22 *tRNA* genes, two *rRNA* genes, and one control region. The gene arrangement was exactly accord with other Lucanidae species. The six PCGs (ND2, ATP6, ND1, ND3, ND5, and ND4) were started with initiation codon ‘ATA’, and four PCGs (COX3, ND4L, ND6, and CytB) were ‘ATG’. The rest of three PCGs (COX1, COX2, and ATP8) have ‘ATT’ as initiation codon. The five PCGs (COX1, COX2, COX3, ND5, and ND4) have incomplete stop codon, ‘T—’. Also, complete stop codon ‘TAG’ and ‘TAA’ were showed in four PCGs (ATP8, CytB, ND1, and ND3) and three PCGs (ATP6, ND4L, and ND4), respectively. The overall PCGs nucleotide compositions were 36% A, 31.8% T, 20.5% C, and 11.7% G with a strong bias toward A＋T (67.8%).

The nucleotide dataset for phylogenetic analysis included 13 PCGs of 23 Lucanidae species. Four Scarabaeidae species was used as outgroup. The maximum-likelihood analysis with 1000 replication of rapid bootstrapping options was performed by PhyML version 3.1 with TVM + I + G model (Guindon et al. 2010).

In phylogenetic analysis, *D. koreanus* was distinguished well in species-level, and *D. koreanus* was grouped with the congeneric species in tribe Dorcini. Also, in case of the species which has a taxonomical confusion, such as *Serrognathus platymelus*, it showed meaningful result for reestablishing their classification through having a specific phylogenetic position in Dorcini group (Figure 1). This result contributes to develop the exact phylogenetic positions of each Dorcini based on morphological and molecular taxonomical data.

**Figure 1.**
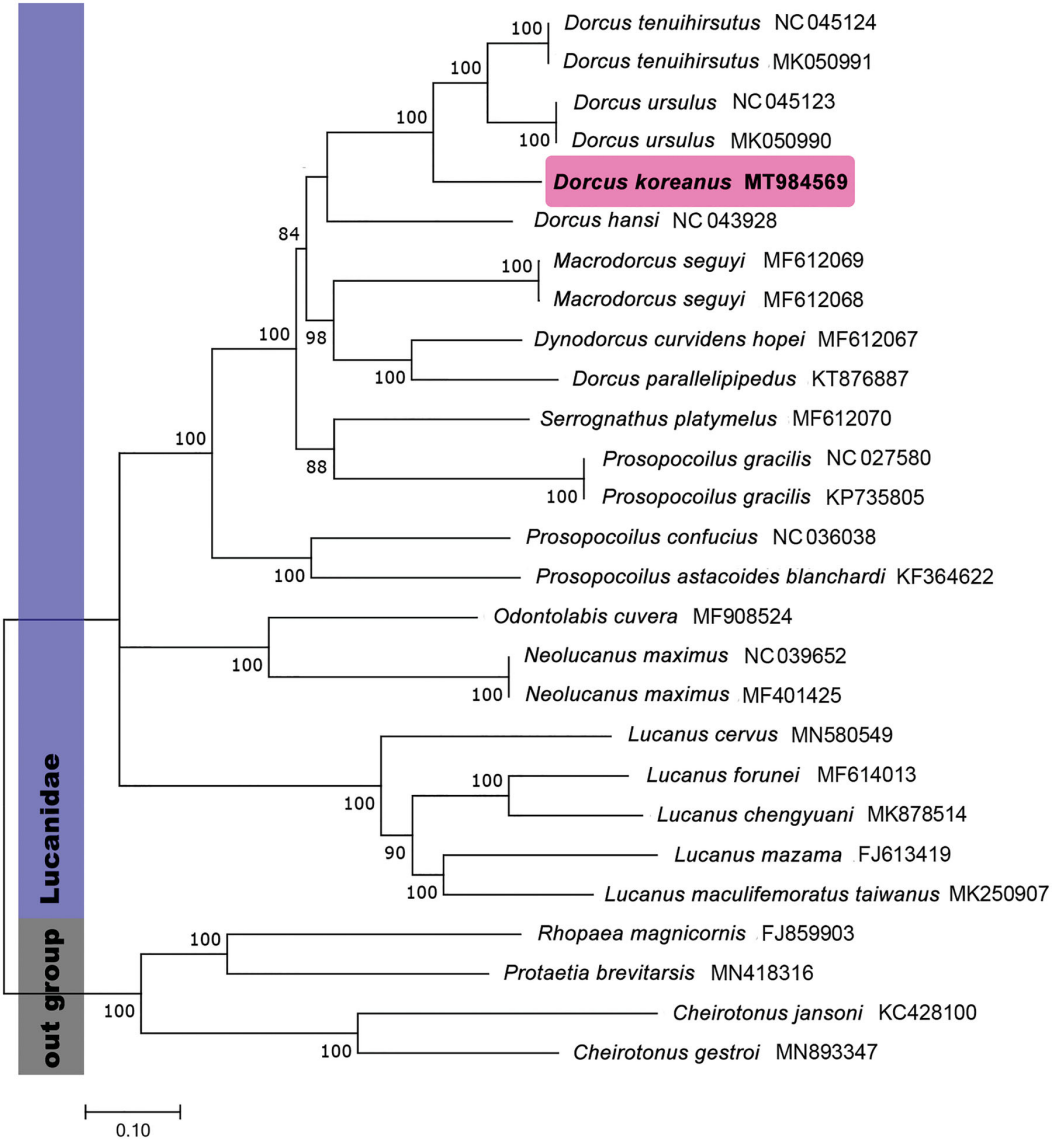
Maximum-likelihood (ML) tree based on analysis of 13 protein-coding genes of 23 mitogenomes of Lucanidae species with TVM + I + G model and bootstrapping (1000 replication).

## Data Availability

The data that support the findings of this study are openly available in GenBank of NCBI at https://www.ncbi.nlm.nih.gov, reference number MT984569.
